# Knowledge Preserving OSELM Model for Wi-Fi-Based Indoor Localization

**DOI:** 10.3390/s19102397

**Published:** 2019-05-25

**Authors:** Ahmed Salih AL-Khaleefa, Mohd Riduan Ahmad, Azmi Awang Md Isa, Mona Riza Mohd Esa, Yazan Aljeroudi, Mohammed Ahmed Jubair, Reza Firsandaya Malik

**Affiliations:** 1 Broadband and Networking (BBNET) Research Group, Centre for Telecommunication and Research Innovation (CeTRI), Fakulti Kejuruteraan Elektronik dan Kejuruteraan Komputer (FKEKK), Universiti Teknikal Malaysia Melaka (UTeM), Hang Tuah Jaya, Durian Tunggal 76100, Melaka, Malaysia; riduan@utem.edu.my (M.R.A.); azmiawang@utem.edu.my (A.A.M.I.); 2 Institute of High Voltage and High Current (IVAT), School of Electrical Engineering, Faculty of Engineering, Universiti Teknologi Malaysia (UTM), Skudai 81310, Johor Bharu, Malaysia; monariza@utm.my; 3 Department of Mechanical Engineering, International Islamic University of Malaysia (IIUM), Selangor 53100, Malaysia; yazan.aljeroudi@gmail.com; 4 Faculty of Computer Science and Information Technology, Universiti Tun Hussein Onn Malaysia, Batu Pahat 86400, Johor, Malaysia; almohamadi611@gmail.com; 5 Faculty of Computer Science, Universitas Sriwijaya (UNSRI), Inderalaya, Sumatera Selatan 30151, Indonesia; rezafm@unsri.ac.id

**Keywords:** fingerprint, indoor localization, learning, Wi-Fi, extreme learning machine

## Abstract

Wi-Fi has shown enormous potential for indoor localization because of its wide utilization and availability. Enabling the use of Wi-Fi for indoor localization necessitates the construction of a fingerprint and the adoption of a learning algorithm. The goal is to enable the use of the fingerprint in training the classifiers for predicting locations. Existing models of machine learning Wi-Fi-based localization are brought from machine learning and modified to accommodate for practical aspects that occur in indoor localization. The performance of these models varies depending on their effectiveness in handling and/or considering specific characteristics and the nature of indoor localization behavior. One common behavior in the indoor navigation of people is its cyclic dynamic nature. To the best of our knowledge, no existing machine learning model for Wi-Fi indoor localization exploits cyclic dynamic behavior for improving localization prediction. This study modifies the widely popular online sequential extreme learning machine (OSELM) to exploit cyclic dynamic behavior for achieving improved localization results. Our new model is called knowledge preserving OSELM (KP-OSELM). Experimental results conducted on the two popular datasets TampereU and UJIndoorLoc conclude that KP-OSELM outperforms benchmark models in terms of accuracy and stability. The last achieved accuracy was 92.74% for TampereU and 72.99% for UJIndoorLoc.

## 1. Introduction 

In this present era of mobile technologies, a broad range of emerging and innovative applications are adopted to enhance communications. Most of these applications connect individuals digitally and are used in transmitting and receiving data via access to a secure cloud environment or to an internal device [1]. Location data allow for the utilization of various services used by individuals and are important in monitoring and tracking devices. The global positioning system (GPS) is a sensing system used to conduct localization analysis. However, GPS signals transmitted via satellites have low power, thereby resulting in weak or no signal when operating in indoor environments. Therefore, constructing systems that can provide acceptable position estimations within indoor environments is crucial. Such systems are referred to as indoor positioning system (IPS). Services that are reliant on IPS are called indoor location-based services (ILBSs) [2]. Examples of these services include ILBSs for tourists who require tools or location guiding services to locate important but unfamiliar places of interest [3]. Another example is observed in large shopping malls and arcades [4], where users typically want to quickly find the location of a shop or a restaurant that has an excellent user rating. In medical facilities and hospitals, the location of wandering patients must be monitored in real time to locate them quickly and provide them with timely treatment. In distribution warehouses, staff must acquire tracking services in place to enable them to perform real-time detection of goods and inventory. In art exhibitions, visitors may view the most intriguing paintings within the enormous compounds in the museum. GPS is not guaranteed to function appropriately in complex indoor areas because satellite signals are often blocked or too weak to be detected. Compared with outdoor environments, high location resolutions are required to determine the position of users in indoor environments [2]. This requirement poses new challenges in system designs for indoor localization based on time-critical constraints with demands for high accuracy and energy efficiency. Innovative developments in wireless and mobile technology have started a new age for indoor localization. Wi-Fi localization, which is based on the sensing and computing of smart devices, is a promising localization technology at present. The reason is that Wi-Fi infrastructure is available in nearly every indoor environment, and Wi-Fi sensing functionality is available in smartest devices.

Machine learning models are widely used for building Wi-Fi-based indoor localization. The model is trained on a fingerprint, which refers to the dataset of Wi-Fi signals observed from all available APs at different locations and arranged as labeled records with their locations in the environment. Such a dataset is prepared by scanning the Wi-Fi signal in the environment in equally distributed locations and labeling such locations. The machine learning model is supposed to be trained on the collected dataset in a process called training or learning. The model is supposed to operate while a person navigates in the environment by using Wi-Fi sensor to measure the Wi-Fi signal again and to consult the trained model regarding its current location. This process is called testing from the perspective of machine learning. In Wi-Fi localization, learning and testing are performed in an online mode because using already known locations with their measured Wi-Fi signal is efficient for adding knowledge to the model or what is denoted technically as online learning. Although online learning is supposed to update the knowledge of the classifier, knowledge loss can still occur. Knowledge update can lead to knowledge loss in dynamical scenarios. A common example of a dynamical scenario that leads to knowledge loss during online learning is the cyclic dynamic behavior. For indoor navigation, cyclic dynamic behavior occurs when a person visits the same place more than once. Most indoor navigation behaviors of people have cyclic dynamic tendency. For example, employees usually visit the same offices on working days because of the job nature. People also usually go to food courts to eat and then return to their working locations. For Wi-Fi-based localization, various models are used or adopted such as Bayesian [5], support vector machine [6], neural network [7], Markov [8], decision tree [9], and extreme learning machine (ELM) [10] models. However, all these models have not tackled the navigation behavior of people in an indoor environment and have not utilized such behavior for achieving improved location prediction results. This study aims to tackle the cyclic dynamic behavior in indoor localization and to develop a machine-learning model based on online sequential ELM (OSELM) for exploiting this behavior in achieving improved prediction results. OSELM is selected because fast learning and testing requirements of indoor localization add advantages to the shallow and efficient models in calculations, such as ELM [11,12].

The remainder of the article is structured as follows. Section 2 discusses the related works. Section 3 provides the background of the study. Section 4 presents the methodology, whereas Section 5 discusses the experimental work and results. Section 6 provides the conclusion and findings of the study.

## 2. Related Works

In the broader scope, various simple approaches were proposed to solving Wi-Fi based localization using created fingerprint for the site. A good example is k-nearest neighbor or KNN [13] where the KNN algorithm starts by calculating the P-norm of M dimensions RSSI vector. Then, it selects the minimum Euclidian distance of the k-neighbor points. Finally, the location is estimated by calculating the average of the coordinates of the k-nearest neighbor. However, such approach assumes a linear relation between the positions and their corresponding RSS signals which is not applicable in practical scenarios. As a result, researchers have suggested neural network NN based approaches where the knowledge pattern that associates between the various locations in the environment and the measured RSS signals of APs at those locations can be captured through training.

One of the most attractive NN models is ELM. In [12], there was a proposal for a new neural network version known as ELM. In [14], the authors demonstrated that ELMs are able to accomplish minimum training error. Liang et al. [15] proposed an online learning mechanism that one can utilize for ELM. In online sequential learning, one can update a base learner using small quantities of incoming data. By using small chunks at a time to learn, a base learner that has undergone previous training can update its knowledge regarding the new data. To manage the practical side of learning, the development of model transfer learning was done for ELM. In [16], there was a proposal for a new cross-domain network learning framework that is based on ELM. This is called the ELM-based domain adaptation (EDA), EDA makes it possible to learn an ELM classifier and a category transformation with random projection through the minimization of the $l_{2,1}$-norm of learning errors and network output weights simultaneously. Unlabeled target data that is considered a useful knowledge are also incorporated as a fidelity term to ensure stability during cross-domain learning. This way lessens the matching error between base and learned classifiers, such that one can readily incorporate numerous existing classifiers as base classifiers. The weights of the network output weights are transferable and can be analytically determined. It also incorporated a manifold regularization with Laplacian graph to facilitate semi-supervised learning. In [17], there was a proposal for a unified framework called domain adaptation ELM (DAELM). In order to learn a robust classifier, the DAELM leverages a limited amount of labelled data from the target domain for gas recognition and for drift compensation in E-nose systems, without losing the learning ability and computational efficiency of traditional ELM. However, online transfer learning is not a part of this approach, which is needed in a broad range of real-world applications. In [18], there was a proposal for a blind domain adaptation algorithm. This algorithm has no need for target domain samples for training. It utilizes a global nonlinear ELM model from the source domain data in an unsupervised manner. It then uses the global ELM model to learn and initialize class-specific ELM models based on the source domain data. During testing, the reconstructed features from the global ELM model are used to augment the features of the target domain. It then classifies the resulting enriched features using the class-specific ELM models, which use the minimum reconstruction error as their basis. Ref. [19] proposed an on-line approach to quickly adapt a “black box” classifier to a new test dataset without the need to retrain the classifier or investigate the original optimization criterion. In this approach, there is an assumption that the original classifier outputs refer to a continuous number where the class is given by threshold. It classifies the points near the original boundary using a Gaussian process regression scheme. One can utilize this general procedure in the context of a classifier cascade. It can also obtain results better compared to the state-of-the-art results obtained through face detection on a standard dataset.

For indoor localization performed with ELM, various researchers have used OSELM for Wi-Fi localization. OSELM is known to have a fast-learning speed that can lessen the time and manpower that are often related to the offline site survey. OSELM is also equipped with an online sequential learning ability that makes it possible for the proposed localization algorithm to quickly and automatically adapt to the environmental dynamics. It was proven by the authors in [20] that OSELM performs better than the batch ELM [12] for indoor localization. Weighted ELM was also incorporated with signal tendency index to perform Wi-Fi-based localization based on the standardized fingerprint. In [21], there was a proposal for two robust ELMs (RELMs), close-to-mean and small-residual constraints, to address issues of noisy measurement in IPSs. There is a determination of the existence of explicit feature mapping in ELM. It then utilizes second-order cone programming to offer kernelised RELM formulations and random hidden nodes. The methods were utilized for indoor localization through Wi-Fi. It provides better accuracy compared to the basic ELM model. Apart from using ELM in Wi-Fi-based localization, a number of researchers, such as those in [22], also made use of it in personal dead reckoning (PDR)-based localization. During the first stage, they formulated the PDR localization process to serve as an approximation function. They then formulated a sliding window-based scheme to pre-process the gathered inertial sensor data and produce the feature dataset. Lastly, they suggested using the OSELM-based PDR algorithm for managing localization issues of pedestrians. OSELM has the ability to adapt dynamically to the localization environment and reduce the localization errors to a lower scale as a result of its universal approximation capability and extreme learning speed. In [23], the researchers proposed an incremental learning model, using transfer learning; MFA-OSELM. In this study, the authors applied the concept to Wi-Fi navigation and showed good performance improvement in the context of feature adaptability of Wi-Fi positioning system to preserve the knowledge in its neural network. The work conducted in [24,25] describes a novel type of extreme learning machine, using external memory and transfer learning; ITM-OSELM. In this study, the authors applied the concept to Wi-Fi localization and showed good performance improvement in the context of cyclic dynamic and feature adaptability of Wi-Fi navigation. However, the approach has used external memory, which means this approach cannot preserve the knowledge in its neural network without the use of external memory. In [10], the researchers used ELM for transfer learning framework. The developed framework has the ability to remove or add APs to the environment, which produces changes in the fingerprint model. Transfer learning is used in neural networks for adopting to a new situation without having to gather the new fingerprint again. One can move the old information that was gathered within the neural network to the new network with the help of two matrices: The input-weight supplement vector and the input-weight transfer matrix. The former helps the system take on the needed adjustments about the changing dimensions of feature matrices among the domains in conjunction with online sequential learning. One can use this model to avoid exhausting and traditional training processes when an expected update happens in the distribution of data because of changes in the domain or the environment. However, a disadvantage of the model is that it loses all old information and knowledge gathered from the network. This knowledge is vital when a high dynamical change takes place in the environment, which helps restore old knowledge in the system in the case of the occurrence of another change. One particular example involving Wi-Fi localization is when users go back and forth within indoor environment areas.

In general, the machine learning developed for Wi-Fi localization takes advantage of the online learning power of transfer learning and ELM models to easily perform efficient operation and training of the model. However, all current models do not take into account a vital feature of indoor navigation, which is its cyclic dynamic behavior. Taking advantage of this behavior can significantly enhance performance. This study emphasizes on this topic.

## 3. Background

This section provides the needed background for the developed methodology. The ELM model is reviewed in Section 3.1. The online variant of ELM or OSELM is provided in Section 3.2. The procedure for each of the two models is also provided. Additionally, feature adaptive-OSELM (FA-OSELM) is presented in Section 3.3.

### 3.1. ELM Review

**Figure 1:**
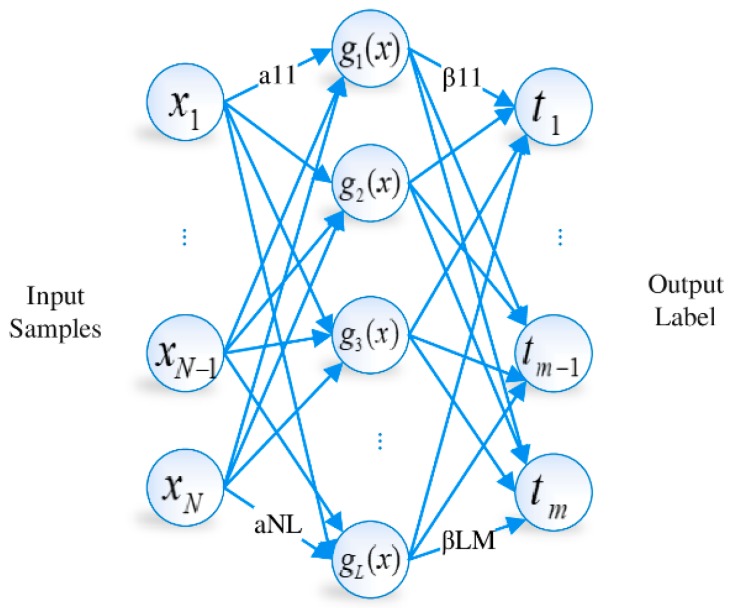
Single hidden layer feedforward neural network used for extreme learning machine (ELM) training.

In [12], a new neural network version called ELM was developed. Figure 1 illustrates the structure of ELM. This new learning framework is considered a single layer feed-forward network (SLFN) that performs random selection of input weights and an analytical determination of output weights through a Moore–Penrose generalized inverse. Algorithm 1 presents the algorithm involved in training an ELM model. This algorithm has attracted broad attention from researchers because it possesses the power of universal approximation and the ability of faster learning than other techniques.


**Algorithm 1: Extreme Learning Machine (ELM) Algorithm**
**Input:**$\mathrm{Training}\;\mathrm{set},\;N=\left\{\left(x_{i},t_{i}\right)\right\}\;|x_{i}\in R^{n},t_{i}\in R^{m},i=1,2,\ldots ,N,\;$activation function g(x)$,\;\mathrm{number}\;\mathrm{of}\;\mathrm{neurons}\;\mathrm{in}\;\mathrm{the}\;\mathrm{hidden}\;\mathrm{layer}\;N_{h}$ **Output:** Output weights, β $1:\;\mathrm{Random}\;\mathrm{values}\;\mathrm{are}\;\mathrm{selected}\;\mathrm{for}\;\mathrm{input}\;\mathrm{weights}\;w_{i}$$\mathrm{and}\;\mathrm{bias}\;b_{i}$, $i=1,2,\ldots ,N_{h}$ 2: The hidden layer output matrix, *H*, is calculated and defined as: (1)$$H={\left[\begin{array}{ccc} g\left(w_{1}x_{1}+b_{1}\right) & \cdots & g\left(w_{N_{h}}x_{1}+b_{N_{h}}\right)\\ \begin{array}{c} g\left(w_{1}x_{2}+b_{1}\right)\\ \cdots \end{array} & \begin{array}{c} \cdots \\ \cdots \end{array} & \begin{array}{c} g\left(w_{N_{h}}x_{2}+b_{N_{h}}\right)\\ \cdots \end{array}\\ g\left(w_{1}x_{N}+b_{1}\right) & \cdots & g\left(w_{N_{h}}x_{N}+b_{N_{h}}\right) \end{array}\right]}_{N\times N_{h}}$$ 3: Output matrix β is computed as(2)$$H^{†}={\left(H^{T}H+\frac{1}{C}\right)}^{-1}H^{T}\;\beta =H^{†\;}T$$ $\mathrm{where},\;H^{†\;}$ represents the Moore–Penrose generalised inverse of *H* that results into the $\mathrm{minimisation}\;\mathrm{of}\;\mathrm{the}\;L_{2}$
normfor both ‖Hβ−T‖ and ‖β‖. C denotes the regularization parameter that is added to prevent the case of singularity. *T* stands for the training set’s label matrix that can be defined as(3)$$T={\left[\begin{array}{c} t_{1}^{T}\\ t_{2}^{T}\\ \begin{array}{c} \vdots \\ t_{N}^{T} \end{array} \end{array}\right]}_{N\times m}$$ where m refers to the dimension of labels that corresponds to every training sample 4: **return**
β


### 3.2. OSELM Review

There are no available data in advance for a wide range of applications. Instead, there is a continuous generation of data based on time. Thus, every time there is a new block available, there is a need to train on the block of data. In [26], there was a development of a mathematical method for conducting online sequential learning for ELM, referred to as OSELM. OSELM includes two major phases. In the boosting phase, training of SLFNs is performed using the primitive ELM method as well as a few batches of training data that were utilized in the initialization stage. Once the boosting phase is complete, it discards the boosting training data. Then, the training data is learned by the OSELM one by one or chunk by chunk. After the learning procedure is performed on these data, it discards all the training data. Algorithm 2 depicts the process of the OSELM algorithm. The symbols of both Algorithm 1; Algorithm 2 are explained in Table 1.


**Algorithm 2: ELM (OS-ELM) Algorithm**
**Inputs**$ℵ=\left\{\left(X_{i},t_{i}\right)|X_{i}\in R^{n},t_{i}\in R^{m},i=1,\ldots ,\tilde{N}\right\}$ **Output** trained SLFN **step 1 Boosting Phase:** $\mathrm{Assign}\;\mathrm{arbitrary}\;\mathrm{input}\;\mathrm{weight}\;W_{i}$$\;\mathrm{and}\;\mathrm{bias}\;b_{i}$$\;\mathrm{or}\;\mathrm{center}\;\mu_{i}$$\;\mathrm{and}\;\mathrm{impact}\;\mathrm{width}\;\sigma_{i},i=1,\ldots ,N.$ $\mathrm{Calculate}\;\mathrm{the}\;\mathrm{initial}\;\mathrm{hidden}\;\mathrm{layer}\;\mathrm{output}\;\mathrm{matrix}\;H_{0}={\left[h_{1,\ldots ,}h_{\tilde{N}}\right]}^{T}$, where $$h_{i}={\left[g\left(W_{1}\cdot X_{i}+b_{1}\right),\ldots ,g\left(W_{\tilde{N}}\cdot X_{i}+b_{\tilde{N}}\right)\right]}^{T},i=1,\ldots ,\tilde{N}.$$ $\mathrm{Estimate}\;\mathrm{the}\;\mathrm{initial}\;\mathrm{output}\;\mathrm{weight}\;\beta^{\left(0\right)}=M_{0}H_{0}^{T}T_{0}$$,\;\mathrm{Where}\;M_{0}={\left(H_{0}^{T}H_{0}\right)}^{-1}$ and $$T_{0}={\left[t_{1},\ldots ,t_{\tilde{N}}\right]}^{T}.$$ Set k=0. **step 2 Sequential Learning Phase:** $\mathrm{For}\;\mathrm{each}\;\mathrm{further}\;\mathrm{coming}\;\mathrm{observation}\;(X_{i},t_{i}$$),\;\mathrm{where},\;X_{i}\in R^{n},t_{i}\in R^{m}$ and $i=\tilde{N}+1,\tilde{N}+2,\tilde{N}+3,\ldots ,$ do $\mathrm{Calculate}\;\mathrm{the}\;\mathrm{hidden}\;\mathrm{layer}\;\mathrm{output}\;\mathrm{vector}\;h_{\left(k+1\right)}={\left[g\left(W_{1}\cdot X_{i}+b_{1}\right),\ldots ,g\left(W_{\tilde{N}}\cdot X_{i}+b_{\tilde{N}}\right)\right]}^{T}.$ $\mathrm{Calculate}\;\mathrm{latest}\;\mathrm{output}\;\mathrm{weight}\;\beta^{\left(k+1\right)}$ based on RLS algorithm:(4)$$M_{k+1}=M_{k}-\frac{M_{k}h_{k}+h_{k+1}^{T}M_{k}}{1+h_{k+1}^{T}M_{k}h_{k+1}}\;\beta^{\left(k+1\right)}=\beta^{\left(k\right)}+M_{k+1}h_{k+1}\left(t_{i}^{T}-h_{k+1}^{T}\beta^{\left(k\right)}\right)\;\mathrm{Set}\;k=k+1$$

### 3.3. FA-OSELM Review

FA was developed by [9] and put forward this model that aims to transfer weight values from the old to the new classifier. Thus, FA-OSELM can be defined as a method of transferring previous knowledge from a pre-trained neural network to a new network, based on the difference in the number of features in both.

Considering that hidden nodes (L) is the same between two networks, FA-OSELM provides an input-weight supplement vector Qi as well as an input-weight transfer matrix P, which allow moving from the old weights $a_{i}$ to the new weights $a_{i}^{\prime }$ with regards to the equation that accounts for the change in the amount of features from $m_{t}$ to $m_{t+1}$:

(5) $${\left\{a_{i}^{\prime }=a_{i}\cdot P+Q_{i}\right\}}_{i=1}^{L}$$

where:

(6) $$P={\left[\begin{array}{ccc} P_{11} & \cdots & P_{1m_{t+1}}\\ \vdots & \ddots & \vdots \\ P_{m_{t}1} & \cdots & P_{m_{t}m_{t+1}} \end{array}\right]}_{m_{t}\times m_{t+1}}$$

(7) $$Q_{i}=\left[\begin{array}{ccc} Q_{1} & \cdots & Q_{m_{t+1}} \end{array}\right]1\times m_{t+1}$$

where matrix *P* must adhere to the following rules:

- For every line, there is only one ‘1’; the rest of the values are all ‘0’;
- Every column has at most one ‘1’; the rest of the values are all ‘0’;
- $P_{ij}=1$ signifies that following a change in the feature dimension, the *i*th dimension of the original feature vector will become the *j*th dimension of the new feature vector.

When the feature dimension increases, $Q_{i}$ will function as the supplement. It also adds the corresponding input-weight for the newly added attributes. Furthermore, the rules below apply to $Q_{i}$:

- Lower feature dimensions indicate that $Q_{i}$ can be termed as an all-zero vector. Hence, no additional corresponding input weight is required by the newly added features;
- In cases where the feature dimension increases, if the new feature is embodied by the *i*th item of $a_{i}^{\prime }$, a random generation of the *i*th item of $Q_{i}$ should be carried out based on the distribution of $a_{i}$.

## 4. Methodology

This section presents our developed methodology for building knowledge preserving OSELM (KP-OSELM). We start with presenting four types of dynamical scenarios that can be regarded as primary scenarios for cyclic dynamic behavior. Then, we present the neural network structure of KP-OSELM and its evolution with respect to time. Thereafter, we introduce our general algorithmic procedure of KP-OSELM. Finally, we provide the evaluation measures for investigating the performance of the developed model by comparison with other state-of-the-art models.

### 4.1. Generating Dynamic Scenarios of Localization

The goal of this subsection is to generate dynamic states related to a person moving in an area from one place to another, which causes a change in available APs. Dealing with mobility scenarios requires emphasis on the aspect of knowledge preservation. For example, a person may move from area A where only N1 features or APs are available to area B where N2 features are available. Then, the person returns to area A, which requires using old knowledge. The work of FA-OSELM has transferred the knowledge from A to B, but knowledge loss may occur due to the change in the dimension of the neural network when the person moves to a less AP area. When the person returns to the old area, the neutral network of the newly transferred knowledge cannot return the lost knowledge. Therefore, an extended period is required to gain the old lost knowledge. This problem can be resolved by developing the concept of knowledge transferring to a new model that assures knowledge preservation with a minimum amount of knowledge loss. To quantify this certain limitation of FA-OSELM.

The following scenarios are made.

**Figure 2:**
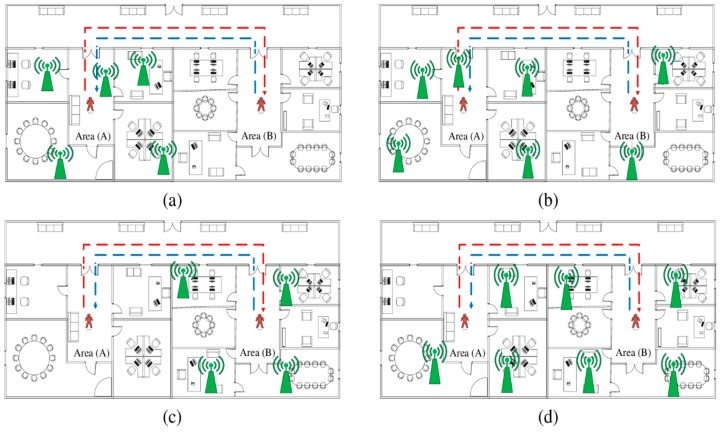
Evaluation scenarios of the different areas in the environment according to APs distribution. (**a**) Scenario 1, $AP_{B}\subset AP_{A}$ and $N_{B}\leq N_{A}$, APs are located in the A side only; (**b**) scenario 2, $AP_{B}⊄AP_{A}$, and $N_{B}\leq N_{A}$, APs are located in both sides with higher density in the A side; (**c**) scenario 3, $AP_{A}\subset AP_{B}$ and $N_{B}\leq N_{A}$, APs are located in the B side only; (**d**) scenario 4, ${\mathrm{AP}}_{A}⊄{\mathrm{AP}}_{B}$, and $N_{A}<N_{B}$, APs are located in both sides with higher density in the B side.

1. A person moves from area A to area B. The number of APs in A ($N_{A}$) is higher than the number of features in B ($N_{B}$). The APs in B are contained in the APs in A or $AP_{B}\subset AP_{A}$. Then, the person returns to area A as represented in Figure 2a. The red line represents the trajectory from A to B, whereas the blue line presents the trajectory from B to A. All APs are in side A, such that $AP_{B}\subset AP_{A}$.
2. A person moves from area A to area B. The number of APs in A ($N_{A}$) is higher than or equal to the number of features in B $(N_{B}$). The APs in B are not contained in the APs in A or $\;AP_{B}⊄AP_{A}$. Then, the person returns to area A as represented in Figure 2b. Similar to the previous scenario, the red line represents the trajectory from A to B, whereas the blue line represents the trajectory from B to A. The APs are distributed on sides A and B with higher density in side A than in side B, such that $N_{A}>N_{B}$ and $AP_{A}⊄AP_{B}$.
3. A person moves from area A to area B. The number of APs in A $(N_{A}$) is lower than the number of features in B $\left(N_{B}\right)$. The APs in B are contained in the APs in A or $AP_{A}\subset AP_{B}$. Then, the person returns to area A as represented in Figure 2c. The APs are distributed on side B only. Thus, the rule of $AP_{A}\subset AP_{B}$ is applied.
4. A person moves from area A to area B. The number of APs in A ($N_{A}$) is lower than or equal to the number of features in B ($N_{B}$). The APs in B are not contained in the APs in A or $AP_{A}⊄AP_{B}$. Then, the person returns to area A as represented in Figure 2d. The APs are distributed on both sides with higher density in side B than in side A. Table 2 summarizes these scenarios.

The procedure of preparing the datasets for such scenarios starts at dividing the original data into two equal parts. The first part indicates the data of area A, whereas the second part indicates the data of area B. Then, random selection is performed to select the features for areas A and B. Each scenario has different selection of B depending on the relation between sets A and B as presented in the scenario description in Table 2. Table 3 shows the pseudocode for the first scenario. A similar approach is done for other scenarios with changing command 7 that refers to the source of preparing the data of area B, either as subset of the data of area A or as intersected set with A.

### 4.2. Neural Network Structure for Knowledge-Preserving Neural Network and Feature Coding

The SLFN structure shown in Figure 1 is used for our NN model. The difference of this structure from classical SLFN for ELM learning is discussed as follows.

1. The number of inputs is variable n, which equals to the number of APs that are sensed in the area. The number of hidden neurons is L, which is determined with the regularization parameter C by using the characterization model (which is built on the basis of the training data).
2. The activation function is tansig
  (8) $$tansig\left(x\right)=\frac{2}{1+e^{-2x}}-1$$

**Figure 3:**
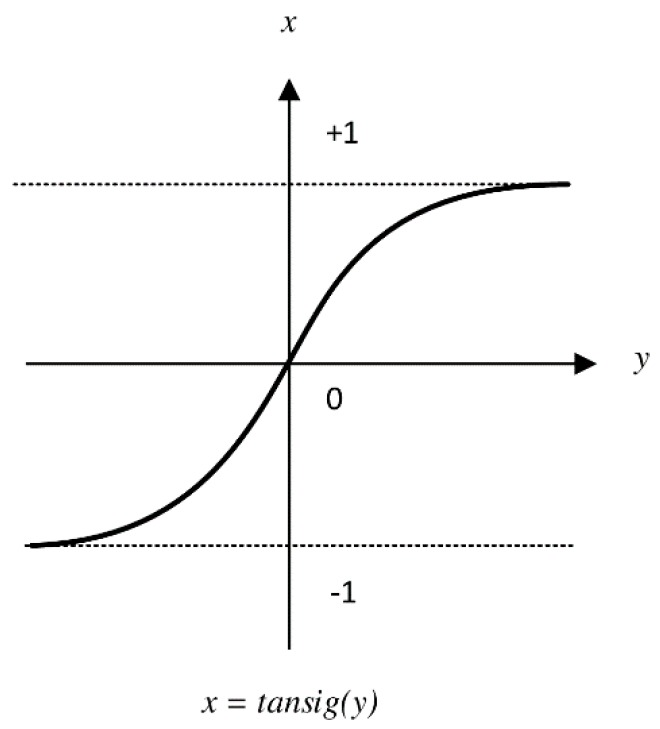
Mathematical curve of tansig function.

This function is selected because it passes through (0,0), which enables the model to cancel the effect of old knowledge (that is gained from non-active APs) because the input is set to 0. Figure 3 presents the mathematical curve of tansig.

**Figure 4:**
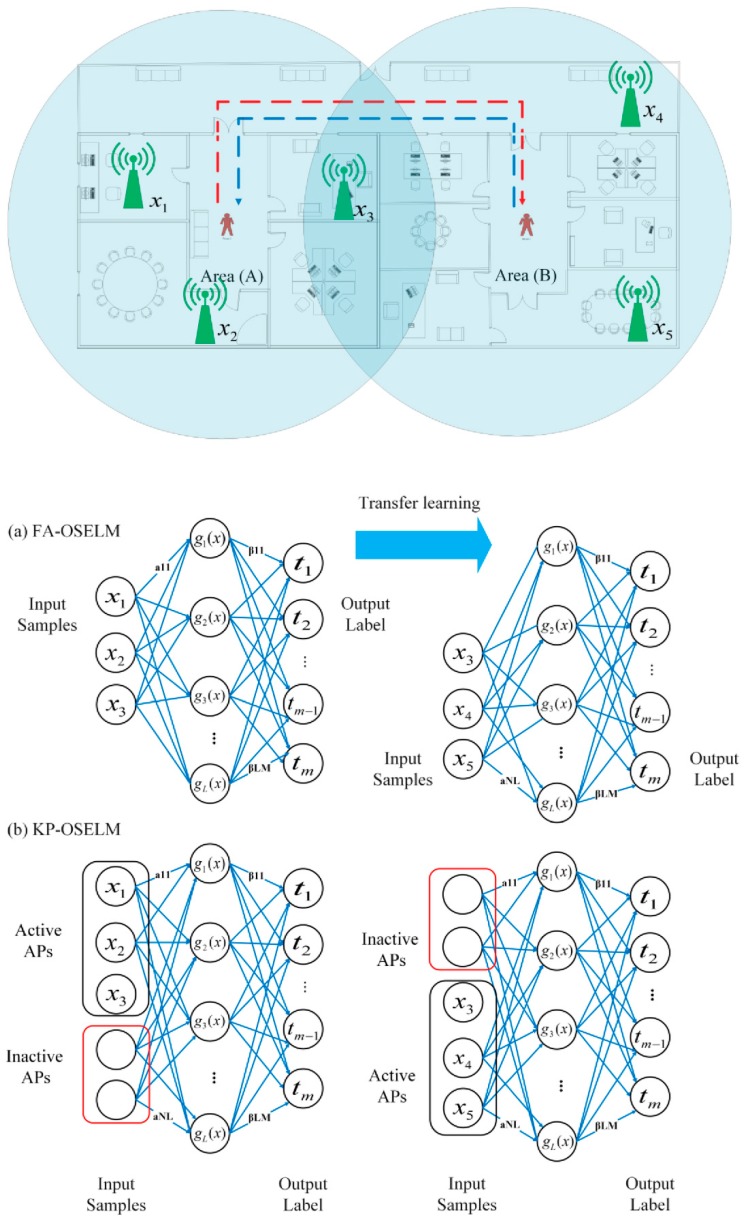
Evolution of single layer feed-forward network (SLFN) according to the change in the number of features (**a**) feature adaptive-online sequential extreme learning machine (FA-OSELM) structure and (**b**) knowledge preserving-online sequential extreme learning machine (KP-OSELM) structure.

Figure 4 depicts the evolution of the neural network structure from one area to another and the APs and their relation with the neural network. Notably, the structure or topology of KP-OSELM does not change similar to that of FA-OSELM, which updates the input number depending on the active features with using a separate transfer learning block to move the needed weights from the old network to the new one as it is shown in Figure 4a. Alternatively, all inputs (active and non-active) in KP-OSELM are kept as it can be seen from Figure 4b. However, the encoding of non-active features with the same value must ensure that the activation functions pass through (0,0). Thus, we use zero encoding for the current tansig. The goal is to cancel the effect of the features in the network decision when they are non-active.

For further elaboration, we consider that the shown NNs are corresponding to a practical example of a person moving from location 1 until location 4 passing through 2 and 3. The APs reading are shown in Table 4. We assume that the non-active APs are encoded with zeros. Thus, when predicting using KP-OSELM, the neural network will receive the input [X_1_ X_2_ X_3_ X_4_ X_5_] = [30 50 60 0 0] when the person was in location 1. Then, it will receive the input [35 45 55 0 0] when the person has moved to location 2. On the other side, in FA-OSELM, NN will have only 3 inputs, and it will receive the vector [X_1_ X_2_ X_3_] = [30 50 60] when the person was in location 1, and [35 45 55] when the person has moved to location 2. Furthermore, when the person moves from location 2 to 3, the structure of KP-OSELM will not change while the structure of FA-OSELM will change to have different inputs [X_3_ X_4_ X_5_] = [70 30 25] in location 3.

### 4.3. General Algorithmic Procedure

KP-OSELM is a novel variant of OSLEM with the capability of preserving the weights of non-active features to restore them when they become active. Its learning equations are similar to the equations of the classical OSELM. The differences are that tansig is used as the objective function and that the constraint of zero features changes within one chunk of data.

The boosting equations are presented as Equations (6)–(9), whereas the iterated equations are presented as Equations (10)–(13). The equations are different from the classical OSELM equations in terms of the input data. In KP-OSELM, we do not use the input vector $X_{i}$. We replace it with vector ${\ddot{X}}_{i}$ that is calculated from Equation (11). This equation indicates that vector ${\ddot{X}}_{i}$ has the same element of $X_{i}$ with active features and has zeros for non-active features. I denotes the active features, whereas F denotes the entire set of features.

(9) $$\beta^{\left(0\right)}=M_{0}H_{0}^{T}y_{0}$$

(10) $$M_{0}={\left(H_{0}^{T}H_{0}\right)}^{-1}$$

(11) $$y_{0}={\left[y_{1},\ldots ,y_{N^{k}}\right]}^{T}$$

(12) $$H_{0}={\left[h_{1,\ldots ,}h_{N^{k}}\right]}^{T}$$

(13) $$h_{i}=[tansig\left(W_{1}\cdot {\ddot{X}}_{i}+b_{1}\right),\ldots ,tansig\left(W_{\tilde{N}}\cdot {\ddot{X}}_{i}+b_{\tilde{N}}\right){]}^{T},i=1,\ldots ,N^{k}.$$

(14) $${\ddot{X}}_{i}=\left(\ddot{x_{j}}\right)$$

$${\ddot{x}}_{j}=\{\begin{array}{c} x_{j}\;\;;j\in I\\ 0;otherwise \end{array}\;\;I=\left\{i_{1},i_{2},\ldots .i_{k}\right\}\subseteq F=\left\{1,\;2\ldots ..n\right\}$$

(15) $$\beta^{\left(k+1\right)}=\beta^{\left(k\right)}+M_{k+1}h_{k+1}\left(t_{i}^{T}-h_{k+1}^{T}\beta^{\left(k\right)}\right)$$

(16) $$M_{k+1}=M_{k}-\frac{M_{k}h_{k}+h_{k+1}^{T}M_{k}}{1+h_{k+1}^{T}M_{k}h_{k+1}}$$

Table 5 provides the pseudocode of learning and prediction, which starts with the boosting phase to train the initial network $SLFN_{0}$ with boosting data $\left(D_{0},y_{0}\right)$. The data contains RSSI information of active features and the corresponding location of any record. Non-active features are encoded with zero values using the encode function. The steps are iterated for as many data chunks are available. Notably, any data chunk contains the same number of active features. We point out that the training and prediction functions adopts the same formulas that are used for OSELM and given in Equations (2) and (4).

### 4.4. Computational Analysis

According to [27], the ELM algorithm has a computational complexity of $O({\tilde{N}}^{2}N)$ where $\tilde{N}$ denotes the number of hidden neurons and N denotes the number of inputs. Since $\tilde{N}$ generally satisfies $\tilde{N}\ll N$ when N is sufficiently large, the computational complexity of the ELM can be thought to approach O(N). For KP-OSELM, we are preserving the structure of ELM with increasing only the dimension of the input vector when the number of inputs increase. Increasing the number of inputs implies only increasing $\tilde{N}$ without changing the number of samples N. As a result, the order of KP-OSELM will stay approximately O(N).

## 5. Experimental Results

This section presents the experimental work for validating KP-OSELM and comparing its performance with those of two approaches, the state-of-the-art approach OSELM [26] and the transfer learning-based approach FA-OSELM [10]. The section is combined of datasets description in Section 5.1. Next, we provide the characterization model in Section 5.2. After that we present the evaluation scenarios. Two approaches of evaluation were used. The first one is the area-based scenario in Section 5.3. This area-based scenario includes the four scenarios that were presented earlier in Section 4.1. The second one is the trajectory-based scenario in Section 5.4.

### 5.1. Datasets Description

The bases of this experiment are the TampereU and UJIndoorLoc databases. The UJIndoorLoc database is made up of three buildings of Universitat Jaume I with areas of almost 110.000 m^2^. These buildings should have at least four levels [28]. One can use this database for classification purposes, such as actual floor and building identification, regression, and actual estimation of latitude and longitude. Development of the database took place in 2013, with more than 20 unique users and 25 Android devices. The database is made up of 19,937 training/reference records and 1111 validation/test records. In 529 attributes, a Wi-Fi fingerprint was observed, which includes the coordinates of where the information was gathered, and other related information.

On the other hand, the TampereU dataset represents an indoor localization database that can be utilized to test IPSs that rely on WLAN/Wi-Fi fingerprint. Lohan and Talvitie created this dataset to test indoor localization techniques [29]. The dataset integrates the two buildings of the Tampere University of Technology. These buildings have three and four levels. The dataset contains 1478 training/reference records and 489 test attributes for the first building, and 312 attributes for the second building. It also contained the coordinates (longitude, latitude, and height) and the Wi-Fi fingerprint (309 WAPs).

An important measure to be considered is the density of APs in each of the two datasets. For TampereU, the density of APs is = number of Aps/area of building = 309/9454 = 0.03 while in UJIndoorLoc the density is the number of Aps/area of building = 520/110,000 = 0.004.

### 5.2. Characterization Model

**Figure 5:**
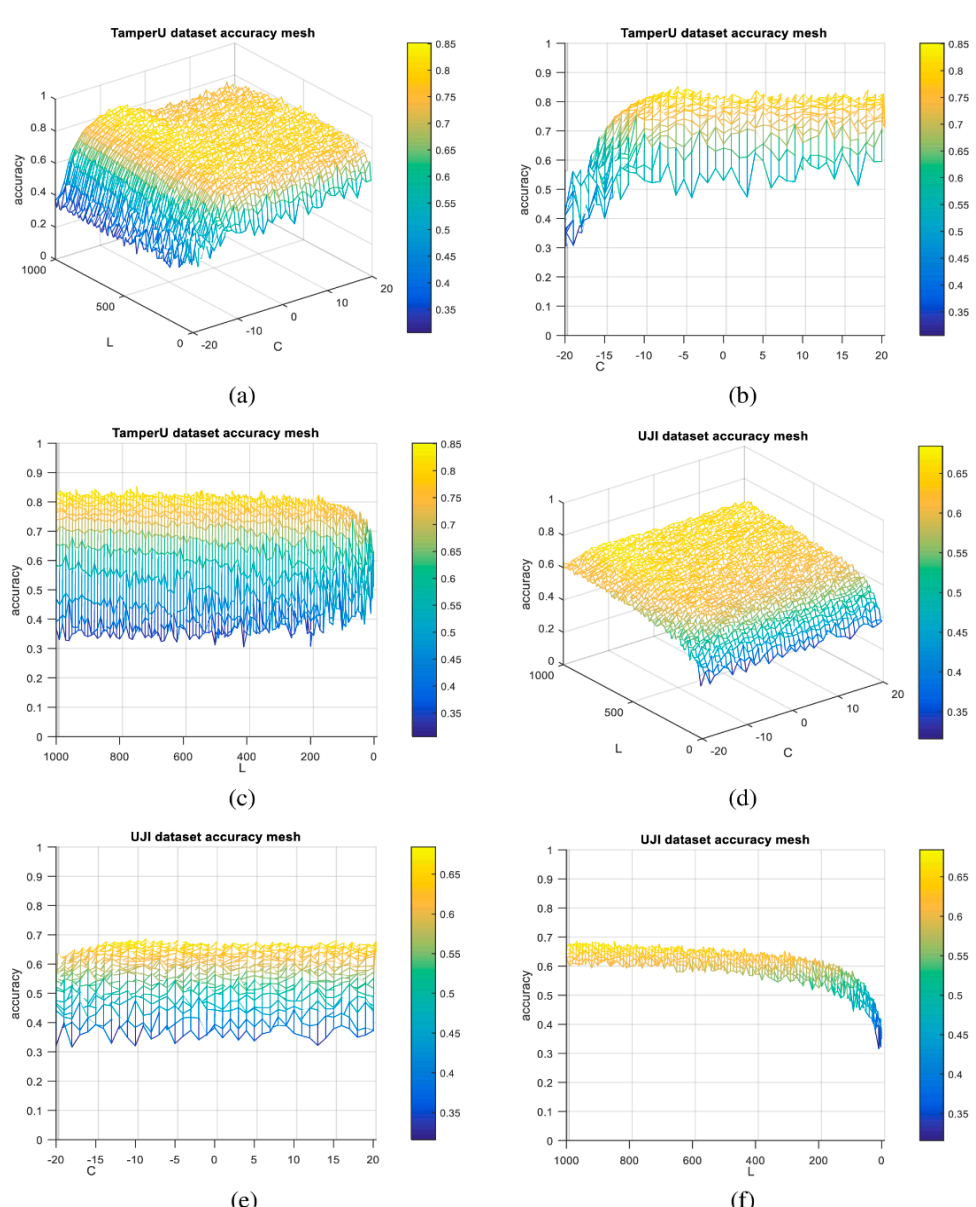
Characterization model of the accuracy of KP-FA-OSELM model according to L and C. (**a**) Mesh representation of the relation between the accuracy, L, and C for ELM classifier based on TampereU dataset; (**b**) relation between the accuracy and C for ELM classifier based on TampereU dataset; (**c**) relation between the accuracy and L for ELM classifier based on TampereU dataset; (**d**) relation between the accuracy, L, and C for ELM classifier based on UJIndoorLoc dataset; (**e**) relation between the accuracy and C for ELM classifier based on UJIndoorLoc dataset; (**f**) relation between the accuracy and L for ELM classifier based on UJIndoorLoc dataset.

In the parameter setting, the regularization parameter (C) is selected to be $C=2^{-6}$ for TampereU based on the relationship between accuracy and C as shown in Figure 5a,b. The number of hidden neurons (L) is selected to be L = 750 for TampereU based on the relationship between accuracy and L as shown in Figure 5a,c. Similarly, L is selected as L = 850, and $C=2^{-9}$ for UJIndoorLoc as shown in Figure 5d–f.

### 5.3. Areas Based Scenarios

The generation of the scenarios is based on the parameters presented in Table 6 and Table 7. Different numbers of features are chosen depending on the scenario and the size of the dataset. High numbers are selected for UJIIndoorLoc because the number of its attributes is high.

For evaluation, we generate the measures for each of the presented scenarios. Our observation of the results is also provided with an analysis and interpretation. Accuracy is presented in Section 5.3.1. The goal of accuracy is to differentiate between our developed model and other benchmark models in terms of location prediction performance. For a quantitative summary of the differences, we provide statistical based comparison that is presented in Section 5.3.2. To investigate the stability of the classifier, we present the standard deviation measure provided in Section 5.3.3. Predicted trajectories vs. ground truth are provided in Section 5.4.

#### 5.3.1. Accuracy

The accuracy of KP-OSELM is generated and compared with that of the two state-of-the-art approaches OSELM and FA-OSELM. The comparison is performed with the TampereU and UJIndoorLoc datasets. The chunk represents a block of data received by the classifier for prediction with the correct values used for training for the next chunk and the accuracy represents the complementary of the error ratio to 1. The error is the ratio of the misclassification over the total number of classification as given in Equation (17):

(17) $$accuracy=1-errorrate=1-\frac{number\;of\;misclassification}{total\;numebr\;of\;classification}$$

**Figure 6:**
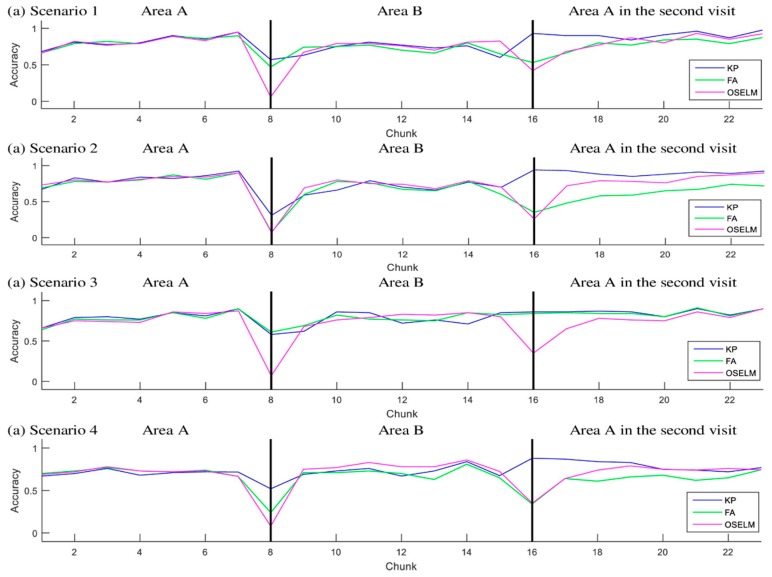
Accuracy for scenarios 1–4 with TampereU dataset.

**Figure 7:**
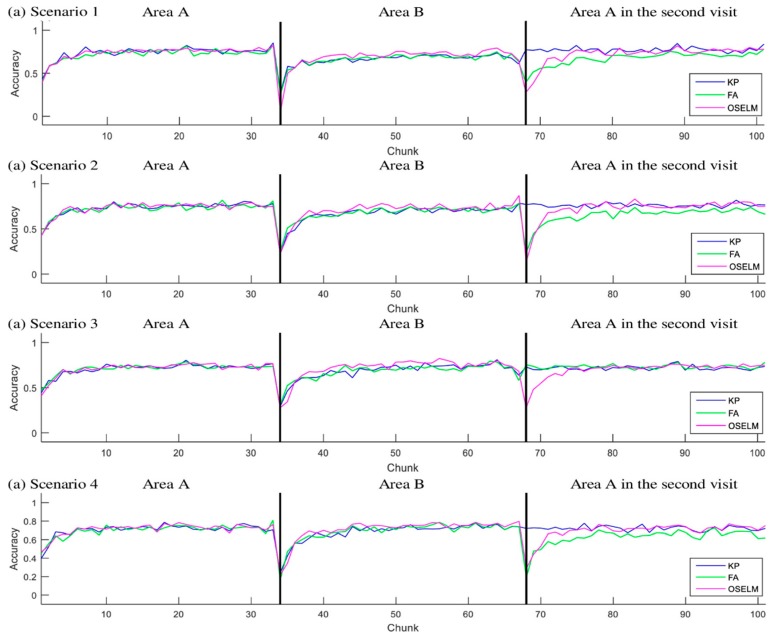
Accuracy for scenarios 1–4 with UJIndoorLoc dataset.

Figure 6 and Figure 7 show the accuracy with respect to the chunks of scenarios 1–4 for the TampereU and UJIndoorLoc datasets, respectively.

In each scenario for the first area, all the classifiers generate similar performance in accuracy. Some tiny changes in accuracy are found, and they are due to the random behavior of ELM. Specifically, the first step in training requires initiating random weights between the input and hidden layer. However, the difference in performance between the approaches occurs when the person leaves an area and enters another area that has been visited earlier. This is interpreted by the fact that the classifier needs to restore an old gained knowledge. The restoration of old knowledge changes between OSELM, FA-OSELM, and KP-OSELM. An example of that is in all scenarios when the subject entered area B, we see that KP-OSELM has achieved an accuracy higher than the two benchmarks FA-OSELM and OSELM. The only similar performance between the benchmarks and KP-OSELM was in scenario 3 because FA-OSELM was able to restore old knowledge because of transfer learning. The exception has occurred in scenario 3 because B is part of A, and the transfer learning has transferred the knowledge of B to A when the subject visited in the second time.

#### 5.3.2. Statistical Based Comparison

In order to verify the improvement of our developed classifier KP-OSELM over the benchmark, we conduct *t*-test to decide when to reject the hypothesis of non-difference. Table 8 and Table 9 present the *t*-test value between KP-OSELM in one side and FA-OSELM and OSELM in the other side for TampereU and UJIndoorLoc, respectively. The t values are found for three areas: A, B, and A for second time. The null hypothesis is rejected for almost all cases of visiting A for the second time by confidence level of 0.1. This emphasizes the claim of superiority of KP-OSELM over both approaches.

#### 5.3.3. Standard Deviation

**Figure 8:**
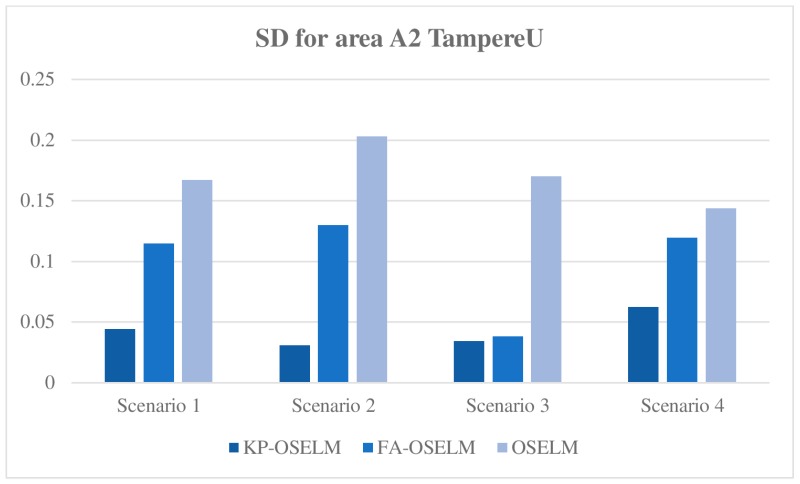
Standard deviation with respect to areas for scenario 1 with TampereU dataset.

**Figure 9:**
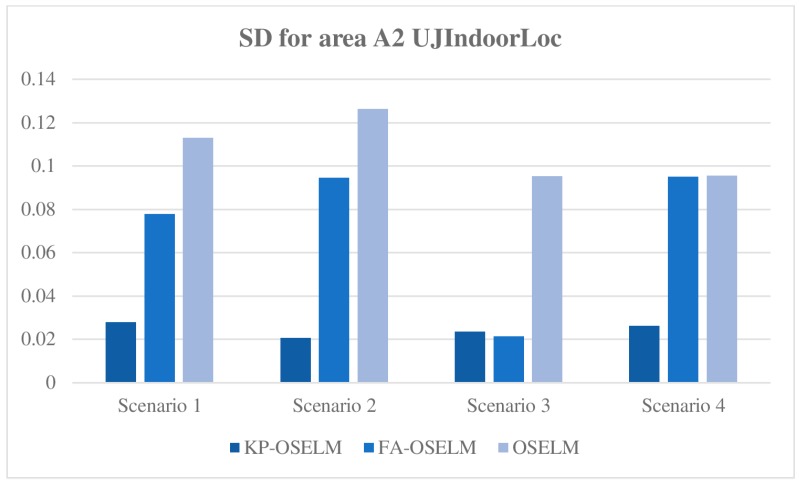
Standard deviation with respect to areas for scenario 1 with UJIndoorLoc dataset.

The standard deviation of accuracy with respect to the chunks of data provides an indicator of the amount of new knowledge in the area that has been gained by the classifier. When a person goes to an early visited area, the classifier may restore the old knowledge. Therefore, gaining any new knowledge is unnecessary or the standard deviation is low, or the knowledge must be gained again because the old knowledge cannot be restored or the standard deviation is high. Figure 8 and Figure 9 show the standard deviation of KP-OSELM, FA-OSELM, and OSELM with respect to the data chunks for A2 with the TampereU and UJIndoorLoc datasets, respectively.

From the figures, the following observations are made. For most scenarios, OSELM has the highest standard deviation because OSELM cannot preserve knowledge when a person goes from one area to another. For all scenarios, KP-OSELM has a small standard deviation or at least equivalent to that of FA-OSELM. By contrast, OSELM has the highest standard deviation in all scenarios. The reason is that OSELM cannot preserve knowledge when the number of features changes, whereas FA-OSELM can transfer knowledge whenever a feature change occurs. However, FA-OSELM has limited capability in knowledge transferring because it can transfer knowledge only from the last state to the current state, whereas KP-OSELM can transfer knowledge from any previous states. In scenario 3 with both datasets, the standard deviation of FA-OSELM is similar to that of KP-OSELM because area A in this scenario is visited from area B and features of area A are part of features of area B. As a result, FA-OSELM can transfer all needed knowledge in area A, which provides FA-OSELM a performance equivalent to that of KP-OSELM.

### 5.4. Trajectory Based Scenarios

The previous evaluation (areas based scenarios) lacks the ability of evaluating the localization algorithm based on given navigation trajectories. The reason is that the testing captures the accuracy of predicting the location of the testing dataset which is composed of different records, however, it does not include an actual records generated from a traversed scenario. In order to test the algorithm based on given scenarios we adopt the simulator that is presented in [24]. This simulator allows providing a whole trajectory and it generates its corresponding time series of records from the dataset. Floor one was considered for generating the trajectory for rectangle and floor one and two were considered for generating the trajectory for cubic. Each of the trajectories was generated with three cycles.

**Figure 10:**
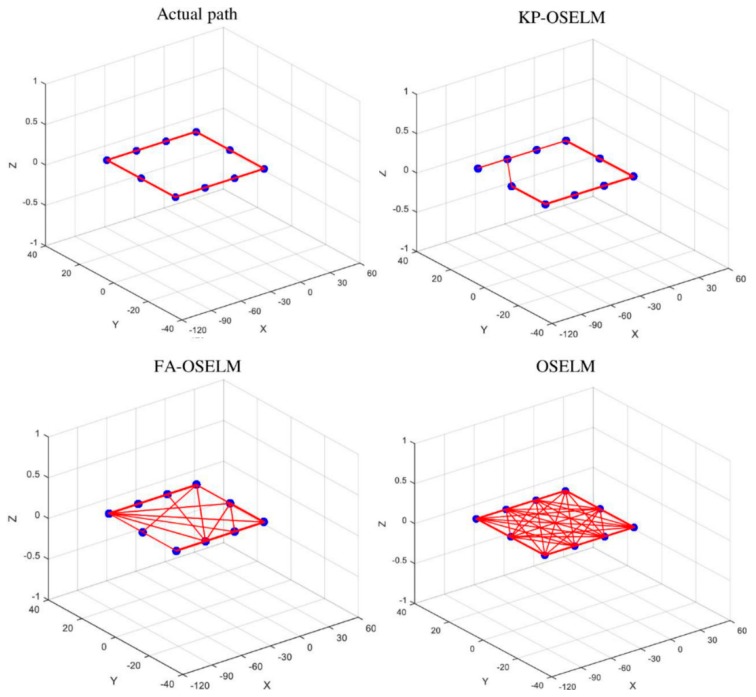
Rectangular trajectory, KP-OSELM, FA-OSELM, and OSELM compared to ground truth for TampereU dataset.

**Figure 11:**
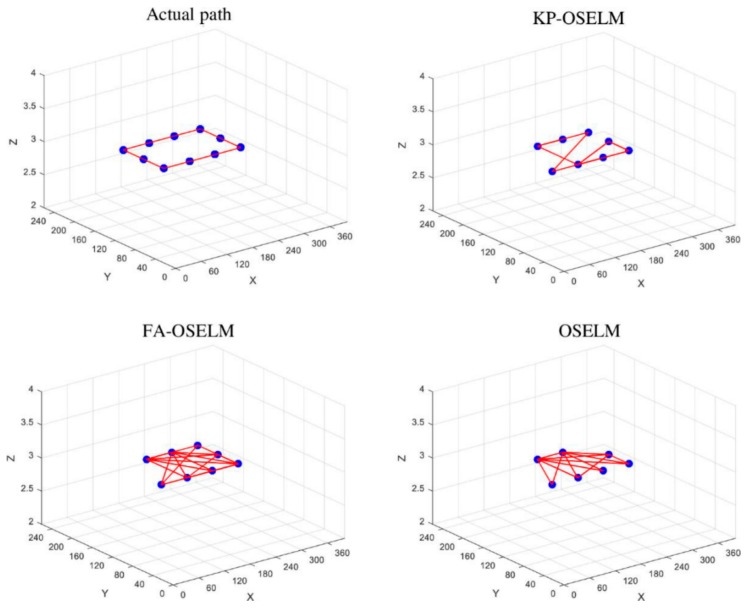
Rectangular trajectory, KP-OSELM, FA-OSELM, and OSELM compared to ground truth for UJIndoorLoc dataset.

The geometry of the trajectories was compared with the ground truth in order to investigate the correctness of the prediction from localization perspective. Ground truth is the actual conducted path by the navigating person. Figure 10 and Figure 11 shows the predicted paths for KP-OSELM, FA-OSELM, and OSELM. In addition, the ground truth path or actual path is shown as the rectangular trajectory for both TampereU and UJIndoorLoc datasets. We represented the paths in graphs were each node in the graph provides one predicted location while the edge represents the previous location that was predicted before the person goes to the current predicted place. We call irregular edges, which are defined as the edges that connect between two nodes that were not connected in the ground truth graph. While missing edges are defined as the edges that are missing between two nodes that were connected in the ground truth graph. The best graphs are the ones with lower number of the two types of edges – irregular edges and missing edges. Based on that, it can be observed that KP-OSELM has provided graphs with lower irregular edges and missing edges compared with FA-OSELM and OSELM graphs. Moreover, in KP-OSELM the edges thickness increases when they match their corresponding in the actual conducted graph. Observing the irregular edges in KP-OSELM, it can be interpreted that the multi-path nature of the Wi-Fi signal in the indoor environment is the reason. However, the frequency of the occurrence of such edges is not high compared with FA-OSELM and OSELM. The achieved accuracy in each of the three cycles of the rectangular trajectory was compared between the three approaches in Table 10. It is obvious that our model KP-OSELM has improved its performance from one cycle to the other. In the third cycle, the accuracy of KP-OSELM has reached 92.74% compared with 78.28% for FA-OSELM and 7.99% for OSELM in TampereU dataset. Similarly, the accuracy of KP-OSELM has reached 72.99% compared with 38.2% for FA-OSELM and 13.12% for OSELM in UJIndoorLoc dataset.

The reason of the difference in the performance between the two datasets is the difficulty in each of them. The difficulty is assumed to be inversely proportional with the density or the ratio of the number of APs to the area. The higher APs is equivalent to lower difficulty and the bigger area is equivalent to higher difficulty. From Section 5.1, In TampereU we see that Tamper has higher density, which causes lower difficulty and higher accuracy.

## 6. Summary and Conclusions

This article presents an online localization approach based on Wi-Fi technology for a person moving in an indoor environment. It focuses on the cyclic dynamic behavior that exists due to the nature of a person’s mobility in an indoor environment, which requires visiting the same places that have been visited previously. This requires from the neural network to save the old knowledge for future usage. To preserve old knowledge, feature coding based on the used activation function is used. The goal of the coding is to cancel out the contribution of old knowledge that has nothing to do in the current situation. When using the tansig activation function, zero encoding of non-active features is needed. The evaluation performed in this study shows the superiority of the developed KP-OSELM over OSELM and FA-OSELM. The superiority is clear in the accuracy improvement, which supports the effectiveness of KP-OSELM compared with FA-OSELM and OSELM. The improvement has been verified statistically using the *t*-test. A further aspect of performance measurement is the standard deviation of accuracy, which is nearly low in all scenarios for KP-OSELM, thereby proving that this model has greater stability and lesser loss of old knowledge than FA-OSELM. A visualization of predicted trajectories by our approach and comparing it with benchmarks and ground truth supports the improvement. Our evaluation concludes the superiority of KP-OSELM over the benchmarks in terms of knowledge preservation, and stability in performance. The reached accuracy of KP-OSELM in the last cycle for TampereU was 92.74% and for UJIndoorLoc was 72.99%.

In the future study, we aim to investigate the effect of common features between two places on knowledge preservation and generalize this knowledge-preserving approach with cyclic dynamic awareness to other types of learners.

## Figures and Tables

**Table 1:** Symbols and their description.

Symbol	Description	Symbol	Description
N	The number of input	H	Output matrix
$x_{i}$	The record of features	T	Target vectors
$t_{i}$	The target of certain record	m	Number of features
i	An index	k	Index
$b_{i}$	Biases	$M_{k}$	Intermediate matrix
g(x)	An activation function	RLS	Recursive least square
$N_{h}$	Number of neurons in the hidden layer	$w_{i}$	Weights between input hidden layer
β	Weights between hidden output layer	C	Regularization parameter

**Table 2:** Dynamics scenario of localization.

Scenario Name	Mobility	Number of APs Relation	APs Subset Relation
S1	A→B→A	$N_{B}<N_{A}$	$AP_{B}\subset AP_{A}$
S2	A→B→A	$N_{B}\leq N_{A}$	$AP_{B}⊄AP_{A}$
S3	A→B→A	$N_{A}<N_{B}$	$AP_{A}\subset AP_{B}$
S4	A→B→A	$N_{A}\leq N_{B}$	$AP_{A}⊄AP_{B}$

**Table 3:** Pseudocode of dataset preparation for testing scenario 1.

**Input:**
trainD: Raw training dataset of Wi-Fi fingerprint
testD: Raw testing dataset of Wi-Fi fingerprint
A, B: Two locations with common features
NA: Number of features in location A
NB: Number of features in location B
**Output:**
trainDataA
trainDataAf
trainDataB
trainDataBf
testDataAf
testDataBf
**Start:**
1- Replace all 100 in trainD and testD with 0 //preprocessing
2- nData = number of records in trainD
3- number of RecordsA=nData/2
4- testDataA = testD
5- testDataB = testD //testData is the same for location A and B
6- nFeatures = number of features in dataset
7- nFeatures = 1:nFeatures //create vector of features labels 1,2..nFatures
8- featuresA = getRandom(features,NA) //select NA random features from features
9- featuresB = getRandom(featuresA,NB) //select NB random features from featuresA because B is contained in A
10- trainDataA = trainData(1:numberofRecordsA)
11- trainDataB = trainData(numberofRecordsA+1:end)
12- trainDataAf = generateFeaturesData(trainDataA, featuresA)
13- testDataAf = generateFeaturesData(testDataA, featuresA)
14- trainDataBf = GenerateFeaturesData(trainDataB, featuresB)
15- testDataBf = GenerateFeaturesData(testDataB, featuresB)
**End**

**Table 4:** Access points measurement for a simple practical navigation scenario.

X_1_	X_2_	X_3_	X_4_	X_5_	Y
30	50	60	0	0	1
35	45	55	0	0	1
60	10	12	0	0	2
55	14	11	0	0	2
50	20	9	0	0	2
0	0	70	30	25	3
0	0	88	33	30	3
0	0	40	70	60	4
0	0	36	66	59	4

**Table 5:** Pseudocode of training and prediction using KP-OSELM.

**Inputs:**
$D_{k}$ //chunks of data with constraint of FC to be zero when k is constant
$y_{k}$ //vector of labels of chunk $D_{k}$, it is only provided after the neural
network makes the prediction of $D_{k}\;$
$SLFN_{0}$ //initial neural network
**Outputs:**
ACC //accuracy of prediction
**Start:**
1- $x_{0}$=Encode($D_{0}$) //this function encode the non-active features with zeros
2- $SLFN_{1}=$OSELMTrain($SLFN_{0},x_{0},y_{0}$)
3- For k =1 until N
$4-\;x_{k}$=Encode($D_{k}$)
5- ${\hat{y}}_{k}=$Predict($SLFN_{k},x_{k}$)
6- ACC=calculateAccuracy(${\hat{y}}_{k},y_{k}$) 7- $SLFN_{k+1}=$OSELMTrain($SLFN_{k},x_{k},y_{k}$)
**End**

**Table 6:** Settings of testing scenarios for block size = 100, regularization parameter $C=2^{-6}$ with TampereU dataset.

Parameter Name	Parameter Description	Scenario 1	Scenario 2	Scenario 3	Scenario 4
**NA**	Number of features in location A	100	100	50	50
**NB**	Number of features in location B	50	50	100	100
**NCF-AB**	Number of Common features between A and B	50	11	50	15
**L**	Number of hidden neurons	750	750	750	750

**Table 7:** Settings of testing scenarios for block size = 100, regularization parameter $C=2^{-9}$ with UJIndoorLoc dataset.

Parameter Name	Parameter Description	Scenario 1	Scenario 2	Scenario 3	Scenario 4
**NA**	Number of features in location A	300	300	150	150
**NB**	Number of features in location B	150	150	300	300
**NCF-AB**	Number of Common features between A and B	150	99	150	80
**L**	Number of hidden neurons	850	850	850	850

**Table 8:** *T*-test probabilities for comparing KP-OSELM with benchmarks in each area for TampereU dataset.

Scenario No.	Algorithms 1 vs. Algorithm 2	Area A	Area B	Area A2
**Scenario 1**	KP-OSELM vs. OSELM	0.280	0.437	0.086
	KP-OSELM vs. FA-OSELM	0.558	0.530	0.017
**Scenario 2**	KP-OSELM vs. OSELM	0.871	0.906	0.085
	KP-OSELM vs. FA-OSELM	0.383	0.584	0.001
**Scenario 3**	KP-OSELM vs. OSELM	0.190	0.630	0.063
	KP-OSELM vs. FA-OSELM	0.023	0.466	0.066
**Scenario 4**	KP-OSELM vs. OSELM	0.369	0.864	0.156
	KP-OSELM vs. FA-OSELM	0.288	0.195	0.016

**Table 9:** *T*-test probabilities for comparing KP-OSELM with benchmarks in each area for UJIndoorLoc dataset.

Scenario No.	Algorithms 1 vs. Algorithm 2	Area A	Area B	Area A2
**Scenario 1**	KP-OSELM vs. OSELM	0.462	0.004	0.007
	KP-OSELM vs. FA-OSELM	0.000	0.532	0.000
**Scenario 2**	KP-OSELM vs. OSELM	0.650	0.000	0.069
	KP-OSELM vs. FA-OSELM	0.184	0.198	0.000
**Scenario 3**	KP-OSELM vs. OSELM	0.439	0.000	0.175
	KP-OSELM vs. FA-OSELM	0.540	0.690	0.006
**Scenario 4**	KP-OSELM vs. OSELM	0.084	0.000	0.068
	KP-OSELM vs. FA-OSELM	0.550	0.316	0.000

**Table 10:** Detailed achieved accuracies in each cycle for KP-OSELM, FA-OSELM, and OSELM for both TampereU and UJIndoorLoc datasets.

Dataset	Algorithm	Cycle 1	Cycle 2	Cycle 3
**Rectangle TampereU dataset**	KP-OSELM	67.81%	92.74%	92.74%
	FA-OSELM	68.94%	72.33%	78.28%
	OSELM	8.06%	5.74%	7.99%
**Rectangle UJIndoorLoc dataset**	KP-OSELM	48.88%	73.03%	72.99%
	FA-OSELM	31.53%	32.52%	38.2%
	OSELM	15.95%	14.08%	13.12%
